# Comparison of Implant Surgery Methods of Cortical Tapping and Cortical Widening in Bone of Various Density: A Three-Dimensional Finite Element Study

**DOI:** 10.3390/ma16083261

**Published:** 2023-04-21

**Authors:** Yeon-Wha Baek, Young-Jun Lim, Bongju Kim

**Affiliations:** 1 Department of Prosthodontics, Gwanak Center, Seoul National University Dental Hospital, Seoul 08826, Republic of Korea; 2 Department of Prosthodontics and Dental Research Institute, Seoul National University Dental Hospital, School of Dentistry, Seoul National University, Seoul 03080, Republic of Korea; 3 Clinical Translational Research Center for Dental Science, Seoul National University Dental Hospital, Seoul 03080, Republic of Korea

**Keywords:** dental implant, finite element analysis, cortical tapping, cortical widening, bone density

## Abstract

Purpose: The primary stability of a dental implant is critical for successful osseointegration during immediate loading. The cortical bone should be prepared to achieve enough primary stability, but not overcompressed. In this study, we investigated the stress and strain distribution in the bone around the implant induced by the occlusal force applied during immediate loading at various bone densities by the FEA method to compare cortical tapping and widening surgical techniques. Materials and Methods: A three-dimensional geometrical model of a dental implant and bone system was created. Five types of bone density combination (D111, D144, D414, D441 and D444) were designed. Two surgical methods—cortical tapping and cortical widening—were simulated in the model of the implant and bone. An axial load of 100 N and an oblique load of 30 N were applied to the crown. The maximal principal stress and strain were measured for comparative analysis of the two surgical methods. Results: Cortical tapping showed lower maximal stress of bone and maximal strain of bone than cortical widening when dense bone was located around the platform, regardless of the direction of the applied load. Conclusions: Within the limitations of this FEA study, it can be concluded that cortical tapping is biomechanically more advantageous to the implants under occlusal force during immediate loading, especially when the bone density around the platform is high.

## 1. Introduction

Primary stability is critical for successful osseointegration of implants, especially during immediate loading. Implant stability is considered an important factor for the healing process, osseointegration and eventual success of the implant [1,2]. Primary stability is decided by the initial fixation force determined by mechanical properties immediately after implant placement. Secondary stability, on the other hand, is determined by bone formation through biological reactions at the bone–implant interface [3]. The factors influencing implant primary stability can be classified into the quantity and density of bone [4], the surgical technique [5,6,7], and the design and surface of the implant fixture [8,9,10].

Cortical bone is of considerable importance for the primary stability of implants [11,12]. Studies have shown that the presence of crested cortical bones has a beneficial effect on stabilizing the implant from micromotion that deteriorates the stability at the interface between bone and implant in the early stages of bone healing [13]. Mosavar et al. [14] suggested that dense cortical bone, especially the bone adjacent to the first thread, experiences the maximum strain upon insertion and bears maximum compressive force and shear stresses. Engaging the implant threads into the cortical layer seems to allow higher implant stability and minimize micromotion, and possibly bear the concentrated loads in the crestal area. Meanwhile, doubts that overcompression or the induced high stresses during implant placement may provoke early periimplant bone resorption and implant failure are raised if this fixation in the crestal cortical bone remains over time [15,16,17]. The crestal region of an implant, often composed of dense cortical bone with a minimal blood supply, is more susceptible to bone necrosis when excessive pressure is applied during placement. Excessive torque placed on an implant can result in high levels of strain transmitted to the adjacent bone, applying irreversible damage in the form of microcracks and plastic deformation that may induce ischemia with subsequent necrosis or sequestrum formation. The high degree of compression of cortical bone generated by the implant is known to cause cell death and necrosis, and ultimately may lead to bone resorption in the cortical bone layer [18,19]. Therefore, although cortical fixation is crucial in obtaining primary stability, excessive stress on the cortical bone around the implant fixture produced by the torque applied when placing implants can induce bone resorption [19].

To prevent excessive compression on dense cortical bones, precise surgical procedures should be followed in order not to insert the implant with a torque value that exceeds the manufacturer’s recommendations, including adequate irrigation. Additionally, a quarter reverse turn of the implant after insertion can minimize stress on the adjacent bone, especially when using tapered implants [18].

Additional cortical drilling steps have been traditionally utilized to prevent excessive torque values during implant insertion. Brånemark used the countersink drill as a traditional protocol for cortical bone [20]. Pretapping, which prepares the implant thread profile prior to implant placement, has been another method to prevent overcompression of dense cortical bone [18,20,21]. However, it is important to keep in mind that the above surgical methods for preventing excessive pressure were based on the delayed loading protocol. This requires an unloaded healing period of 3–6 months, during which secondary stability increases, and thus sufficient initial stability is not a prerequisite [20,22,23].

In immediate loading protocols that have been widely used in recent years, occlusal force is applied on implants that are not completely osseointegrated. Stress and strain magnitude and distribution induced by occlusal force could have an impact on the stability during healing and the success of the implant. Previous studies have shown that implants placed after cortical drilling or pretapping in low-density bone lack primary stability due to loss of coronal fixation, and may be inadequate for early or immediate loading protocols [4,24].

Therefore, cortical bone should be prepared so that the implant can achieve good primary stability in its crest sufficient to withstand the occlusal load, but not be overcompressed to prevent bone resorption or osteonecrosis [11,12,16]. However, there have been no clear guidelines on how to achieve this optimal fitting in cortical bones [25].

In this study, we investigated the stress and strain distribution in the bone around the implant induced by the occlusal force applied during immediate loading according to surgical methods at various bone densities by the FEA method to compare cortical tapping and widening surgical techniques. The hypothesis of this study was that no significant differences would be found in the stress and strain distribution in the bone around the implant induced by the occlusal force applied between the two surgical methods of cortical widening and cortical tapping.

## 2. Materials and Methods

**Figure 1:**
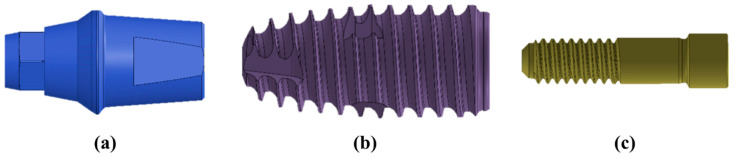
Three-dimensional CAD model of dental implant system used in this study: (**a**) abutment, (**b**) implant fixture, and (**c**) abutment screw.

**Figure 2:**
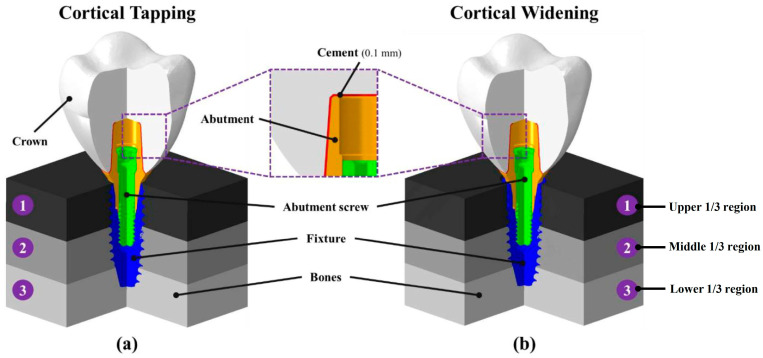
Multi-cross-sectional view of second molar surgical model with two surgical techniques. Each surgical model consists of abutment, fixture, abutment screw, crown, cement and bone. The bone was divided into three regions of ①–③ according to height. (**a**) Surgical model using cortical tapping, (**b**) Surgical model using cortical widening.

The three-dimensional geometrical model of the dental implant and bone system was created using CAD software (Solidworks 2016; Dassault Systemes SolidWorks Corp., Waltham, MA, USA). The design of one dental implant (IS-II; Neobiotec Inc., Seoul, Republic of Korea), which has an S-shaped collar and 0.8 mm thread pitch, was used. An implant fixture of 10 mm length and 4.5 mm diameter, abutment of 8 mm length, and abutment screw of 8.8 mm length and 1.95 mm diameter with a 2.3 mm diameter screw head and 0.4 mm pitch were designed in the software (Figure 1). The monolithic zirconia crown and 0.1 mm cement thickness between the abutment and crown were assumed for prosthesis (Figure 2). The physical properties of each element in the model were adopted with reference to the relevant literature, as shown in Table 1 [26,27,28].

**Figure 3:**
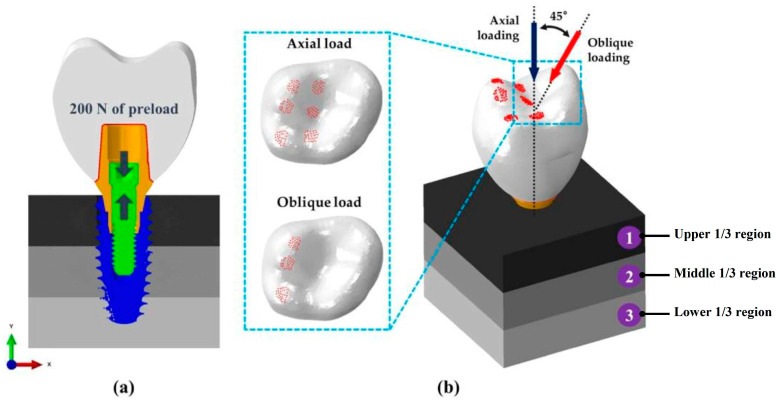
(**a**) 200 N preload of force to tighten the abutment screw to the fixture, (**b**) mastication load applied in axial and oblique (45°) directions. The bone was divided into three regions of ①–③ according to height.

The bone model was simplified in the form of a cuboid of 12 mm in height and 15 mm × 15 mm in width and length. Previous studies have classified the density of the alveolar bone into four categories—D1 to D4 [28,29,30,31,32]. In this study, different-density bones were combined to reproduce situations that can be encountered in actual clinical practice. The bone was divided into three regions according to height, and one bone density among the lowest D1 and highest D4 of bone density was set for each region (Figure 2 and Figure 3). A total of five types of bone density model (D111, D144, D414, D441 and D444) were designed as shown in Table 2.

The two surgical methods of cortical tapping (CP) and cortical widening (CW) were implemented as the FEA models. For the CP model, the thread shape of the implant fixture body was formed inside the bone so that the interface between the bone and the implant was in full contact (Figure 3). In the CW model, bone up to 3 mm deep from the surface was removed in a cylindrical shape with a diameter of 4 mm, which was larger than the implant diameter. From the 3 mm depth of the bone to the fixture tip, the bone was removed in the form of the fixture shape (Figure 3).

A finite element analysis (FEA) program (ABAQUS CAE2016; Dassault Systems, Vélizy-Villacoublay, Yvelines, France) was used to construct assembled models for each component of the dental implant system (fixture, abutment, abutment screw), crown, cement, and bone. The mesh of the FEA model was formed using Hypermesh software (Altair Hypermesh v19.0; Altair Engineering, Troy, MI, USA). With reference to previous studies, the mesh size of the bone was set within a minimum of 0.15 mm to a maximum of 0.75 mm, and that of the implant system was set from a minimum of 0.03 mm to a maximum of 0.15 mm. From 0.15 mm to 0.3 mm and from 0.03 mm to 0.1 mm were set for the mesh sizes of the crown and the cement, respectively [26,33]. Information on elements, nodes, and mesh sizes used in the present study are shown in Table 3.

A preload of 200 N was applied to the abutment screw as a force to tighten the abutment screw to the fixture (Figure 3). To reproduce the vertical and lateral force applied on the teeth during mastication, an axial load of 100 N and an oblique load of 30 N at 45 degrees [34,35,36] were applied in this study (Figure 3). An axial load of 100 N was applied to three cusps and three fossa corresponding to 187 nodes, and a 45-degree tilted load of 30 N was applied to the three cusps through 86 nodes. The side and bottom surfaces of the bone were fully constrained against motion and rotation in the three axes of x, y, and z. A “tie contact” was applied to the interface between the bone surrounding the fixture and the fixture in consideration of initial fixation. The interfaces between the inner surfaces of the crown, cement and the abutment were constructed to be perfectly bonded. On the other hand, a coefficient of friction of 0.5 and the “surface-to-surface” contact condition were considered between the components of the dental implant system (fixture, abutment, and abutment screw) [37].

The maximal principal stress and maximal principal strain were measured for comparative analysis of the two surgical methods. A higher principal stress and strain value means a higher risk of bone fracture and implant fixation failure. Peak von Mises stress (PVMS) was measured to compare the failure risk of the implant systems, crown and cement, for the two surgical methods.

## 3. Results

**Figure 4:**
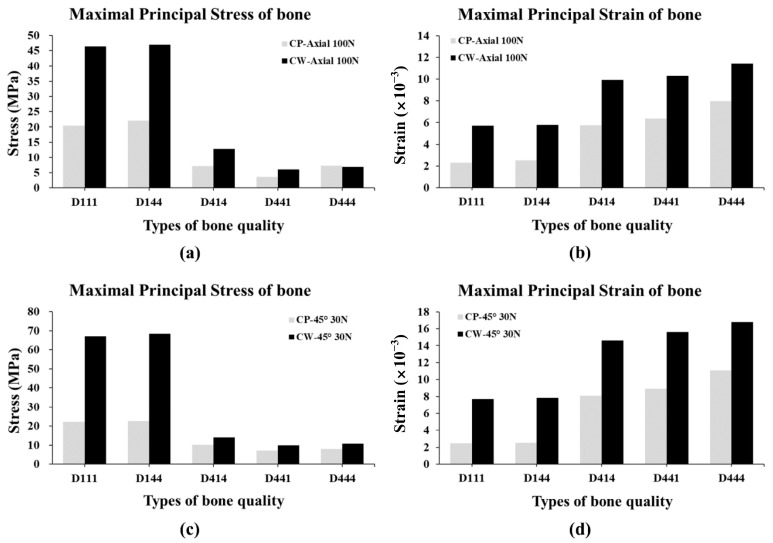
Maximal principal stress and maximal principal strain values in the 5 types of bone with 2 surgical methods. (**a**) Maximal principal stress, (**b**) strain values under 100 N axial load, (**c**) maximal principal stress, and (**d**) strain values under 30 N oblique (45°) load.

The maximal principal stress and maximal principal strain values in the five bone situations with the two surgical methods are shown in Figure 4.

The maximal principal stress of CW group under axial loading was at least 64.7% and up to 126% higher than the CP group in all types of bone, except in the D444 bone (Figure 4a) (Table 4). The maximal principal strain of the CW group under axial loading was higher than the CP group in all bone qualities, with a minimum of 43.4% and a maximum of 149.4% higher bone deformation compared with the CP group (Figure 4b) (Table 4). The CW group also showed higher stress under the 45-degree tilted oblique load in all bone densities, with a minimum of 34.8% and a maximum of 202.9% higher values compared with the CP group (Figure 4c) (Table 4). Similarly to the maximal principal stress results, the CW group showed more bone deformation than the CP group in all type of bone density. The strain of the CW group was at least 51.4% and up to 213.5% higher than the CP group (Figure 4d) (Table 4).

**Figure 5:**
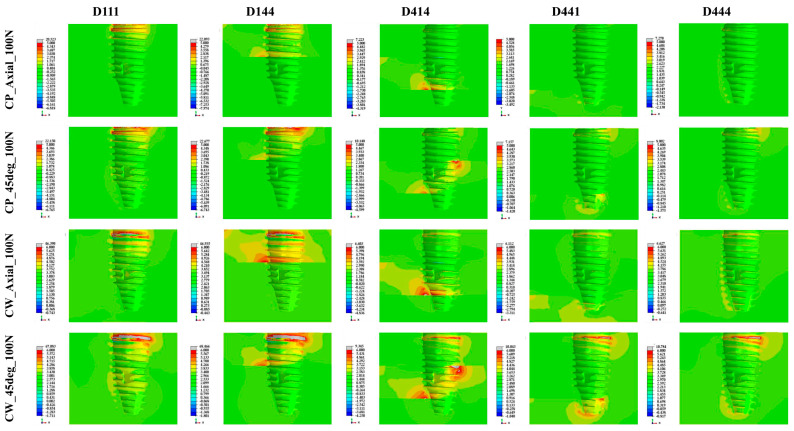
Cross-sectional view of the maximal principal stress values for the 5 bone densities and 2 surgical techniques under 100 N axial load and 30 N oblique load.

**Figure 6:**
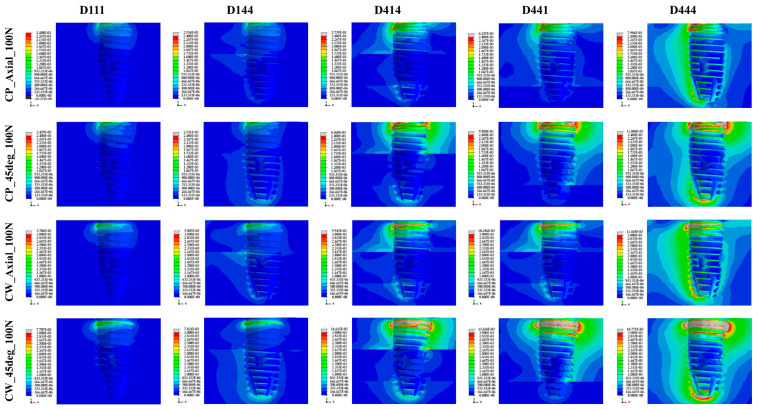
Cross-sectional view of the maximal principal strain values for the 5 bone densities and 2 surgical techniques under 100 N axial load and 30 N oblique load.

The stress and strain of the bone were higher when the load was applied in the oblique direction than in the axial direction, and this was more evident in the CW group than in the CP group (Figure 4) (Table 3). Cross-sectional views of the stress and strain values for five different bone density and two different surgical techniques under 100 N axial load and 30 N oblique load are shown in Figure 5 and Figure 6.

The CW group showed a wider area with stress of 6 MPa or more under the same load and bone-quality conditions than the CP group (Figure 5). As for the strain, the CW group also showed higher strain and a wider area of high strain than the CP group around the platform and the tip of the fixture (Figure 6).

**Figure 7:**
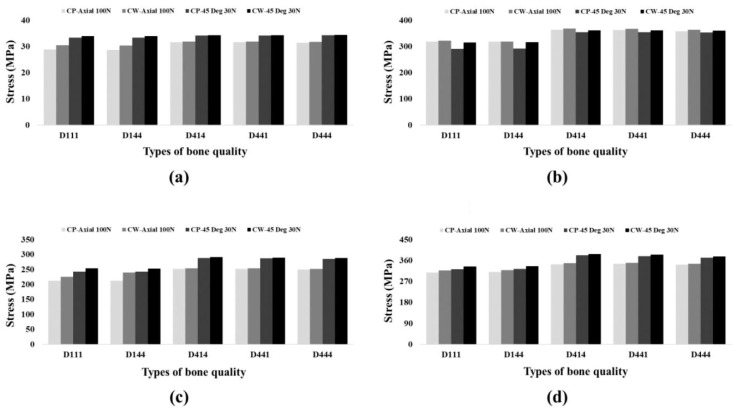
The PVMS of each component for the 5 bone densities and 2 surgical techniques under 100 N axial load and 30 N oblique load. (**a**) PVMS of crown, (**b**) PVMS of abutment, (**c**) PVMS of fixture, and (**d**) PVMS of abutment screw.

To analyze the failure risk of the dental implant system and crown for the two surgical methods in different bone densities, the PVMS values for each component (crown, abutment, fixture, and abutment screw) were measured, and are shown in Table 5 and Figure 7.

The CW group showed higher stresses at the dental implant system (abutment, fixture and abutment screw) and the crown than the CP group (Table 5) (Figure 7). The stress on the abutment under the oblique direction load decreased compared to the vertical direction load. The stress applied to the fixture and abutment screw increased as much as the stress applied to the abutment decreased. On the other hand, when the load was applied in the axial direction, the stress applied to the abutment increased and the stress applied to the fixture and abutment screw decreased (Table 5) (Figure 7). The PVMS values of the cement showed no difference between the CW and CP groups, and those under the oblique direction were higher than under the vertical direction.

## 4. Discussion

We focused on the relationship between the surgical methods and stress and strain distribution on different bone density conditions when occlusal force was applied during healing period before complete osseointegration. We compared two surgical techniques—cortical tapping and cortical widening—as methods of preventing overcompression of the bone. Cortical tapping is a method of preparing the implant thread profile into the recipient bed of the cortical bone before implant insertion to allow pressure-free seating. To pretap, especially on the crestal cortical bone, the cortical tapping drill is shorter than the implant length, with its upper structure identical to the structure of the coronal part of the implant. Cortical widening, such as countersink drilling, is performed to enlarge the crestal area before implant insertion with a countersink drill, which has slightly larger diameter than the implant diameter to prevent overcompression of the dense cortical bone.

The maximal stress of bone was higher with the cortical widening technique than cortical pretapping, and it was more obvious when a high-density bone was located around the platform of the implant (D111 and D144). Higher stress was observed when oblique load applied than axial load in the cortical widening technique, while no significant difference was observed between oblique and axial loads in the cortical pretapping technique. The reason for this could be that the space was formed around the fixture platform in the CW group and the length of the fixture engaged in the bone without the space was short (Figure 5 and Figure 6). The maximal strain of the bone showed a similar trend. Higher maximal strain of the bone was observed when using cortical widening than cortical pretapping for all bone densities. Higher strain was observed when oblique force applied than axial force in both surgical techniques in all bone densities, except for cortical tapping in the dense bone located around the fixture platform. When dense bone was located around the platform (D111 and D144), the maximal stress and strain of bone with cortical tapping were lower than with cortical widening, regardless of the direction of the applied load. The lower stress and strain of the bone with the pretapping technique can be interpreted such that the fixture installed with this technique could receive lower stress in the surface of the cortical bone around the fixture, resulting in less damage to cortical bone and consequently a higher success rate.

The stress was concentrated in the part with high bone density due to the high modulus of elasticity of the bone. The magnitude of the maximum stress increased significantly when high-density bone (D1) was placed around the implant platform (D111 and D144) than when the density of bone around the implant platform was low (D414, D441, and D444). As for the maximal principal strain of bone, the strain rate was much larger around the platform where the bone density around the platform was low (D414, D441, and D444) compared with when the bone density around the platform was high (D111 and D144). When comparing the stress and strain distribution according to the location of the high-density bone (D144, D414, and D441), it was remarkably observed that the maximum stress was high and maximum strain low when the high-density bone was located around the fixture platform. Similarly to previous literature [34,37,38], our results showed that the bone density around the platform is a critical factor for the stress and strain of bone. High bone density around the platform is advantageous to reduce the strain on the bone, but increases the stress on the bone around the fixture, possibly resulting in overcompression or osteonecrosis. Therefore, a separate cortical preparation could be required for high-density crestal bone to obtain optimal primary stability without jeopardizing the threshold level of irreversible damage.

In this study, cortical tapping and cortical widening techniques were compared as cortical preparation methods. Lower stress and strain on bone was observed with cortical tapping than cortical widening, regardless of the direction of occlusal force in all bone density conditions. The differences in stress and strain of two surgical methods was more obvious when the density of upper bone was high (D111 and D144).

The results of our study support previous research comparing insertion torque values of cortical pretapping and widening (drilling) techniques. In another study, higher insertion torque values were recorded for pretapping than widening, especially for class 4 bone in the presence of a 1 mm of cortical bone layer. The authors concluded that cortical widening presented lower insertion torque, because the cortical drill diameter was slightly larger than the implant diameter and deprived the crestal cortical bone and allowed passive fitting of the implant, but at the same time decreased primary stability. The pretap drill was shorter than the implant length, with its upper structure identical (slightly smaller in diameter) to the structure of the coronal part of the implant [25]. The lower structure of the tap was more sharply tapered and narrower to assist insertion into the undersized channel. In our study, comparing the stress and strain induced by occlusal force during immediate loading on the bone around the fixtures installed by the cortical pretapping and widening techniques, respectively, using the FEA methods, it was similarly concluded that cortical tapping that showed lower stress and strain under occlusal force may be the more advantageous surgical technique to maintain primary stability during healing, especially when the bone density around the platform is high. If the implant is inserted exactly into the thread profile prepared by cortical tapping, in a theoretical point of view, maximum surface contact between the implant and the dense cortical bone will be achieved, thereby being more likely to bear the occlusal load. However, this requires further investigation [25].

In this study, maximal stress of bone and maximal strain of bone was higher in cortical widening at all bone densities, and the difference in stress between the two procedures was larger, especially when the bone density around the implant platform was high. Clinically, in case of high bone density, separate drilling is performed to prevent side effects caused by excessive condensation. Therefore, the results of this study showing that cortical tapping is biomechanically more advantageous than widening may be clinically useful when the bone density is high, at least of the upper part.

## 5. Conclusions

Within the limitations of this FEA study, it can be concluded that cortical tapping is biomechanically more advantageous for implants under occlusal force during immediate loading, especially when the bone density around the platform is high.

## Figures and Tables

**Table 1:** Material properties of the FEA model.

Types	Materials	Young’s Modulus (MPa)	Poisson’s Ratio
Bone	D1	9500	0.3
	D4	690	0.3
Abutment	Ti-grade 5	114,000	0.33
Fixture	Ti-grade 4	105,000	0.34
Abutment Screw	Ti-grade 5	114,000	0.33
Crown	Zirconia	205,000	0.19
Cement	Resin	10,310	0.35

**Table 2:** Classification of bone model: three bone regions with two bone densities.

Types	Region ①	Region ②	Region ③
D111	D1	D1	D1
D144	D1	D4	D4
D414	D4	D1	D4
D441	D4	D4	D1
D444	D4	D4	D4

**Table 3:** Number of nodes and elements and mesh sizes of the components.

Components	Elements	Nodes	Mesh Size (mm)	
			Maximum	Minimum
Bone (widening)	398,016	72,982	0.75	0.15
Bone (tapping)	386,123	71,233	0.75	0.15
Abutment	541,131	118,764	0.15	0.03
Fixture	790,066	146,527	0.15	0.03
Abutment Screw	502,530	93,818	0.15	0.03
Crown	147,556	30,528	0.3	0.15
Cement	42,608	14,282	0.1	0.03

**Table 4:** The maximal principal stress and strain of CP and CW in five bone-quality conditions.

**Type of Loading**	**Bone Quality**	**Maximal Principal Stress (MPa)**	
		**CP**	**CW**
100 N at axial direction	D111	20.52	46.39
	D144	22.09	46.94
	D414	7.22	12.82
	D441	3.71	6.11
	D444	7.27	6.96
30 N at 45 degrees	D111	22.15	67.08
	D144	22.68	68.47
	D414	10.14	14.02
	D441	7.16	10.04
	D444	8.01	10.78
**Type of Loading**	**Bone Quality**	**Maximal Principal Strain (×10^−3^)**	
		**CP**	**CW**
100 N at axial direction	D111	2.29	5.71
	D144	2.54	5.81
	D414	5.74	9.95
	D441	6.35	10.29
	D444	7.98	11.45
30 N at 45 degrees	D111	2.46	7.71
	D144	2.53	7.82
	D414	8.06	14.62
	D441	8.90	15.64
	D444	11.08	16.77

**Table 5:** The PVMS of CP and CW in five bone-quality conditions.

Component	Type of Loading	Bone Quality	PVMS (MPa)	
			CP	CW
Abutment	100 N at axial direction	D111	318.88	321.73
		D144	364.34	319.12
		D414	364.34	368.82
		D441	362.28	367.06
		D444	358.28	364.16
	30 N at 45 degrees	D111	290.47	314.71
		D144	291.85	316.62
		D414	354.94	361.05
		D441	354.51	361.08
		D444	353.53	360.61
Fixture	100 N at axial direction	D111	212.34	225.32
		D144	212.03	239.54
		D414	251.48	253.60
		D441	251.29	253.29
		D444	249.38	251.27
	30 N at 45 degrees	D111	242.29	253.11
		D144	242.42	252.59
		D414	288.21	290.65
		D441	286.75	288.94
		D444	285.27	287.40
Crown	100 N at axial direction	D111	28.84	30.48
		D144	28.66	30.34
		D414	31.66	31.89
		D441	31.59	31.83
		D444	31.45	31.78
	30 N at 45 degrees	D111	33.36	33.90
		D144	33.38	33.91
		D414	34.22	34.30
		D441	34.22	34.31
		D444	34.24	34.35
Screw	100 N at axial direction	D111	307.93	316.36
		D144	309.83	317.9
		D414	343.44	347.47
		D441	346.03	349.76
		D444	341.50	345.22
	30 N at 45 degrees	D111	321.66	333.93
		D144	323.20	334.69
		D414	381.33	387.40
		D441	378.52	384.32
		D444	371.03	376.68

## Data Availability

The data that support the findings of this study are available from the corresponding author upon reasonable request.
